# The effect of *Lactobacillus rhamnosus* hsryfm 1301 on the intestinal microbiota of a hyperlipidemic rat model

**DOI:** 10.1186/1472-6882-14-386

**Published:** 2014-10-10

**Authors:** Dawei Chen, Zhenquan Yang, Xia Chen, Yujun Huang, Boxing Yin, Feixiang Guo, Haiqing Zhao, Tangyan Zhao, Henxian Qu, Jiadi Huang, Yun Wu, Ruixia Gu

**Affiliations:** College of Food Science and Technology, Yangzhou University, Yangzhou, 225127 Jiangsu Province China; Key Lab of Dairy Biotechnology and Safety Control, Yangzhou, 225127 Jiangsu Province China; Royal Dairy (Guangxi) Co., Ltd., Nanning, Guangxi 530007 China

## Abstract

**Background:**

Growing evidence indicates that intestinal microbiota regulate our metabolism. Probiotics confer health benefits that may depend on their ability to affect the gut microbiota. The objective of this study was to examine the effect of supplementation with the probiotic strain, *Lactobacillus rhamnosus* hsryfm 1301, on the gut microbiota in a hyperlipidemic rat model, and to explore the associations between the gut microbiota and the serum lipids.

**Methods:**

The hyperlipidemic rat model was established by feeding rats a high-fat diet for 28 d. The rats’ gut microbiota were analyzed using high-throughput sequencing before and after *L. rhamnosus* hsryfm 1301 supplementation or its fermented milk for 28 d. The serum lipids level was also tested.

**Results:**

The rats’ primary gut microbiota were composed of Bacteroidetes, Firmicutes, Proteobacteria, Spirochaetes and Verrucomicrobia. The abundance and diversity of the gut microbiota generally decreased after feeding with a high-fat diet, with a significant decrease in the relative abundance of Bacteroidetes, but with an increase in that of Firmicutes (*P* < 0.05). Administration of *L. rhamnosus* hsryfm 1301 or its fermented milk for 28 d, could recover the Bacteroidetes and Verrucomicrobia abundance and could decrease the Firmicutes abundance, which was associated with a significant reduction in the serum lipids’ level in the hyperlipidemic rats with high-fat diet induced. The abundance of 22 genera of gut bacteria was changed significantly after probiotic intervention for 28 d (*P* < 0.05). A positive correlation was observed between *Ruminococcus* spp. and serum triglycerides, *Dorea* spp. and serum cholesterol (TC) and low-density lipoprotein (LDL-C), and *Enterococcus* spp. and high-density lipoprotein. The *Butyrivibrio* spp. negatively correlated with TC and LDL-C.

**Conclusions:**

Our results suggest that the lipid metabolism of hyperlipidemic rats was improved by regulating the gut microbiota with supplementation of *L.rhamnosus* hsryfm 1301 or its fermented milk for 28 d.

**Electronic supplementary material:**

The online version of this article (doi:10.1186/1472-6882-14-386) contains supplementary material, which is available to authorized users.

## Background

The total number of microbes in the adult gut is 10^14^, which is 10-fold higher than the number of human body cells [1]. It has been proposed that the capacity of the microbiome largely exceeds the human genome with more than three million genes [2], which are rich in carbohydrates, amino acids, vitamins, and other genes involved in nutrient metabolism, most of which are absent in humans [3]. The intestinal microbes affect our lives not only through food metabolism and exclusion of pathogens, but also by modulating the mucosal immune response [4]. Hence, they have a remarkable potential to influence the physiological functions and health of the host [5, 6].

Probiotics are defined as live micro-organisms that confer health benefits to the gut microbiota of the host when present in adequate amounts. The function of the intestinal microbiota is not understood, yet. Nonetheless, advances in culture-independent molecular techniques have provided insight into the composition of the intestinal microbiota before and after probiotic intake [7].

Elevated serum lipids’ level is widely recognized as a primary risk factor for the development of atherosclerosis, coronary heart disease and other cardiovascular diseases [8]. Currently, drug therapy is the principal treatment, but with high relative costs and side effects, it is not considered to be an optimal long-term treatment. Recent studies have demonstrated that *Lactobacilli* or *Bifidobacteria* could exhibit hypolipidemic effects in animal models [9, 10] and in humans [11, 12]. Furthermore, the intestinal microbiota could improve the host’s lipid metabolism via microbial activities [13]. The widely accepted mechanism is that microbiota activities promote bile acid biotransformation *in vivo* to regulate fat digestion, and affect lipid metabolism to decrease serum lipids [14].

In the present study, *L. rhamnosus* hsryfm 1301, which is a strong hypolipidemic agent *in vitro*, was isolated from the gut of subjects from Bama, Guangxi Province, China, who are known for increased longevity. As dairy products may be suitable vehicles for the delivery of probiotics and may enhance the effect of products on the host’s health, *L. rhamnosus* hsryfm

1301-containing skim milk suspension and its fermented milk were used to investigate the effect on serum lipids and on the composition of the intestinal microbiota in hyperlipidemic rats, and to explore the possible mechanism of hypolipidemic of lactic acid bacteria *in vivo*.

## Methods

### Bacteria and culture

*L. rhamnosus* strain hsryfm 1301 was obtained from the Key Lab of Dairy Biotechnology and Safety Control, Yangzhou University, which was isolated from the gut of subjects from Bama longevity, Guangxi Province, China, in 2013. The bacteria were grown in MRS medium at 37°C in an anaerobic jar (Ruskinn Technologies, Ltd., South Wales, UK) for 24 h.

Cells were collected by centrifugation at 4,000 × *g* for 10 min at 4°C. The *L. rhamnosus* hsryfm 1301-containing skim milk suspension was prepared by suspending the cells in 10% (w/v) sterile skim milk to achieve a viable count of 10^9^ CFU/mL, which was then stored at 4°C. Ten-fold serial dilutions of the suspension were performed, and 100 μL were plated on MRS agar (pH 6.8 ± 0.2) in triplicate. Aerobic plates were placed in an anaerobic jar (Ruskinn Technologies, Ltd.) at 37°C for 48 h. The colonies counted after incubation represented the numbers of *L. rhamnosus* hsryfm 1301.

### Preparation of fermented milk

*L. rhamnosus* hsryfm 1301 was added to 10% heated skim milk to a final concentration of 3% (v/v) inoculum, and fermented at 42°C until the milk curdled. Fermentation was stopped when the bacterial viable count was 10^9^ CFU/mL by bacterial counting as above. The fermented milk was stored at 4°C.

### Animal trial

#### Animal groups and diets

Thirty-eight male Sprague–Dawley (SD) rats, aged 5 weeks and weighing 140 ± 4.5 g were purchased from Comparing Medical Center of Yang Zhou University (Jiangsu, China). The rats were exposed to a 12 h light/ dark cycle, and maintained at a constant temperature of 23 ± 1°C and humidity of 50 ± 5%. The care and use of rats was according to our institutional and national guidelines, and all experiments were approved by the Ethics Committee of the Yang Zhou University.

The rats were subjected to a 1-week adaptation period on a normal diet containing 20% (w/w) flour, 10% rice flour, 20% corn, 26% drum head, 20% bean, 2% fish powder and 2% bone powder (XieTong, Organism Inc., Jiangsu, China), The rats were randomly assigned to four groups, control and model group (11 rats each), and two treatment groups (eight rats each). The initial average body weight was similar among the four groups. The four groups were given the following diets: (1) control group, normal diet; (2) model group, high-fat diet; (3) hsryfm 1301 group, high-fat diet + *L. rhamnosus* hsryfm 1301-containing skim milk suspension; (4) hsryfm 1301-f group, high-fat diet + *L. rhamnosus* hsryfm 1301-containing skim fermented milk. The high-fat diet contained 10% (w/w) lard oil, 1% cholesterol, 0.2% sodium cholate and 78.8% normal diet (XieTong, Organism Inc.). The rats had free access to water and their specific diet. The hyperlipidemic rat model was established by providing a high-fat diet for 28 d. The hsryfm 1301 group and hsryfm 1301-f group received daily 2 mL (10^9^ CFU/mL) of *L. rhamnosus* hsryfm 1301-containing skim milk suspension and *L. rhamnosus* hsryfm 1301-containing skim fermented milk, respectively, which was administered intragastrically for 28 d, after the hyperlipidemic rat model was established The control and model groups received an equivalent volume of saline. Their body weight and food consumption were measured weekly and daily, respectively.

### Sample collection

Three fresh fecal samples of 1.00 g (wet weight) were collected from each group on day 1, 28, and 56 before feeding. Samples were suspended in 15.0 mL of 0.10 mol/L phosphate buffer (pH 7.4) by vortexing for 5 min, followed by addition of 10.0 mL of phosphate buffer and vortexing for 5 min. This procedure was repeated, and then samples were centrifugated at 200 × *g* for 5 min to collect the supernatant (bacteria). The fecal samples were dealt with twice as above to collect the supernatant. The supernatant was centrifuged at 9000 × *g* for 5 min, and then the pellet was suspended in 30.0 mL of phosphate buffer by vortexing for 5 min followed by centrifugation as above. The sediment was collected and suspended in 10 mL of 0.10 mol/L phosphate buffer (pH 7.4) by vortexing for 5 min, aliquoted into five tubes, and kept at −70°C for DNA extraction.

Three rats, which were fasted for 12 h and euthanized, were selected randomly from the control and model group at day 28. Blood samples (4 mL) were collected into nonheparinized vacuum collection tubes from the celiac vein. Tubes were initially held stationary at 0°C for 30 min, and then the serum was separated from the blood by centrifugation at 2, 000 × g for 10 min at 4°C, and was used to analyze the lipids’ level.

### Serum lipids analysis

Triglycerides (TG), total cholesterol (TC), high density lipoprotein cholesterol (HDL-C) and low density lipoprotein cholesterol (LDL-C) in the serum were analyzed by commercial kits (Maker, Biotechnology Inc., Sichuan, China) and the chemical analyzer 7020 (Hitachi, Tokyo, Japan). After administering the treatment intragastrically for 28 d, all rats were weighed and the TC, TG, HDL-C and LDL-C were measured by the above mentioned methods.

### Gut microbiota analysis

Microbial DNA from the fecal samples was extracted using QIAamp DNA stool mini kit (Qiagen Inc., Hilden, Germany) according to the manufacturer’s instructions. The V3 hypervariable region of the 16S rDNA was PCR amplified from the microbial genomic DNA using universal primer (forward primers: 5′-ACTCCTACGGGAGGCAGCAG-3′, reverse primers:

5′- TTACCGCGGCTGCTGGCAC-3′). The PCR condition were 94°C for 4 min, followed by 21 cycles of 94°C for 30 s, 58°C for 30 s (annealing) and 72°C for 30 s (extension), and then 72°C for 5 min.

The PCR products were excised from a 1.5% agarose gel and purified by AxyPrep Gel Extraction Kit (Axygen, Scientific Inc., Union City, CA, USA). They were then quantified by PicoGreen dsDNA Assay Kit (Life Technologies Inc., Grand Island, NY, USA) and BioTek Microplate Reader (BioTek Inc., Winooski, VT, USA). The Barcoded V3 amplicon was sequenced using the pair-end method by Illumina Miseq at Personal Biotechnology Co., Ltd (Shanghai, China). Sequences reads with an average quality score lower than 25, ambiguous bases, homopolymer > 7 bases, containing primer mismatches, or reads length shorter than 100 bp were removed. For V3 pair-end read, only sequences that overlapped more than 10 bp and without any mismatches were assembled [15]. Reads that could not be assembled were removed. Barcode and sequencing primers were trimmed from assembled sequences.

### Statistical analysis

The SPSS software (IBM Corp, Armonk, NY, USA) was used to analyze the serum lipids data and the association between serum lipids and intestinal microbiota.

Sequences were clustered and assigned to operational taxonomic units (OTUs) using the Quantitative Insights into Microbial Ecology (QIIME) implementation of cd-hit according to a threshold of 97% pairwise identity. The OTU of every sample and the number of sequences of every OTU were counted after the OTU output. The taxonomy information of every OTU was obtained by searching the most similar species. The rarefaction curve was generated by OTUs at the 97% similarity cut-off level. Rarefaction analysis was performed in Analytic Rarefaction v.1.3 (Hunt Mountain Software, Athens, GA, USA). The Chao1 and ACE abundance indexes, and the Simpson and Shannon diversity indexes were calculated using Mothur software (http://www.mothur.org/) to analyze Alpha diversity. The SPSS (IBM Corp), Fast UniFrac (http://bmf2.colorado.edu/fastunifrac/) and QIIME (http://qiime.sourceforge.net/) software were used to analyze sequence data, bacterial community distribution and the principal component.

## Results

### Effects of *L. rhamnosus*hsryfm 1301 and its fermented milk on physical indexes of a hyperlipidemic rat model

**Figure 1:**
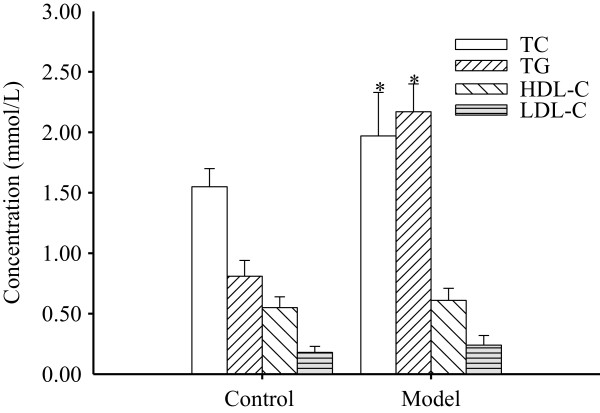
**Serum lipid levels of the control and model groups at 28 d.** Control group: normal diet; model group: high-fat diet. Each concentration is the mean ± standard deviation (n = 3). *, *P* < 0.05 vs. control.

The rats in the control and model groups were fed with normal diet or high-fat diet for 28 d, respectively. The serum lipids’ levels are summarized in Figure 1. The TC and TG levels in the model group were significantly higher than those in the control group (*P* < 0.05; Figure 1), indicating that the hyperlipidemia rat model was successfully established. The HDL-C and LDL-C levels in the control group were lower than those in the model group, but not significantly (*P* > 0.05; Figure 1).

**Figure 2:**
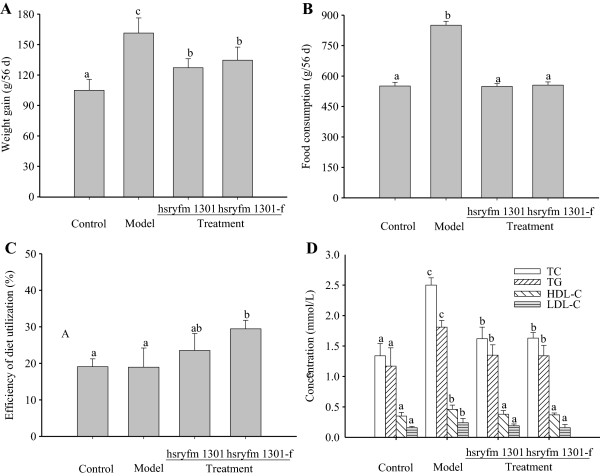
**Physical indexes of rats.** Control group: normal diet; model group: high-fat diet; hsryfm 1301 group: high-fat diet + *L. rhamnosus* hsryfm 1301-containing skim milk suspension; hsryfm 1301-f group: high-fat diet + *L. rhamnosus* hsryfm 1301-containing skim fermented milk. **A**: Weight gain of the rats in 56 d; **B**: Food consumption of the rats in 56 d; **C**: Efficiency of diet utilization of the rats; **D**: Serum TC, TG, HDL-C and LDL-C concentration in the rats. Efficiency of diet utilization (%) = (Weight gain/Food consumption) × 100. Each value is the mean ± standard deviation (n = 8). A different superscript letters means significant difference in the same index (*P* < 0.05).

The rats’ body weight increased during the study period. At the end of the experiment, the gained body weight and the average food consumption of the model group were significantly higher than those of the other groups (*P* < 0.05; Figure 2A and B). The efficiency of diet utilization of the hsryfm 1301-f group was significantly higher than that of the control and model groups (*P* < 0.05; Figure 2C), indicating that the *L. rhamnosus* hsryfm fermented milk improved the efficiency of diet utilization.

After the rats were administered intragastrically *L. rhamnosus* hsryfm 1301 or its fermented milk for 28 d, the TC, TG and HDL-C levels in the control, hsryfm 1301 and hsryfm 1301-f groups were significantly lower than those in the model group (*P* < 0.05; Figure 2D). Also, the LDL-C level in the hsryfm 1301-f group was significantly lower than that in the model group (*P* < 0.05; Figure 2D). The results indicate that *L.rhamnosus* hsryfm 1301 and its fermented milk had obvious effects on rat serum lipids.

### Effects of *L. rhamnosus*hsryfm 1301 and its fermented milk on rats intestinal microbiota

#### Sequencing results of fecal samples

In total, 87,228 sequence reads were obtained and grouped into 8,244 OTUs at the 97% similarity cut-off level. Among the high quality sequences, more than 95% were longer than 146 bp, with most ranging between 146 and 177 bp. The number of OTUs of Phylum, Class, Order, Family and Genus detected by sequencing was 5,359, 4,348, 4,140, 2,583 and 2,583, respectively.

Rarefaction analysis showed that the OTUs of 36 samples gradually increased with the increase of the number of measured sequences. Furthermore, the curve became more gentle and the increasing trend became smaller (see Additional file 1: Figure S1), indicating that most of the bacterial sequences in the samples obtained by the Illumina Miseq Sequencing system reflected the abundance and diversity of the gut microbiota.

#### Analysis of alpha diversity of gut microbiota

The Chao1, ACE and Shannon indexes were significantly higher and the Simpson index was significantly lower at 1 d compared with after feeding high-fat diet for 28 d or normal diet for 56 d (*P* < 0.05; Table 1), indicating that the abundance and diversity of the gut microbiota in rats decreased with the weight increase. However, the abundance and diversity recovered or was even higher than the initial level after rats were administered intragastrically *L. rhamnosus* hsryfm 1301 or its fermented milk for 28 d (*P* < 0.05; Table 1). These results showed that high-fat diet decreased the abundance and diversity of the gut microbiota in rats, while the *L. rhamnosus* hsryfm 1301 or its fermented milk improved them.

**Table 1:** **Alpha diversity of gut microbiota in rats**

Group	Sample collection time (d)	Abundance index		Diversity index	
		Chao1	ACE	Simpson	Shannon
Control	1	16332.081^a^	23580.922^a^	0.067^b^	15.876^a^
	28	16957.731^a^	23883.323^a^	0.072^b^	15.705^a^
	56	14196.252^b^	21689.237^b^	0.088^a^	15.460^b^
Model	1	13796.613^a^	19983.587^a^	0.063^c^	15.864^a^
	28	12538.624^b^	18339.896^b^	0.075^b^	15.221^b^
	56	11200.145^c^	18168.955^b^	0.103^a^	14.828^c^
Hsryfm 1301	1	14699.544^a^	21791.684^b^	0.075^b^	15.531^a^
	28	12836.493^b^	19663.483^c^	0.096^a^	14.763^b^
	56	14947.348^a^	22180.152^a^	0.077^b^	15.455^a^
Hsryfm 1301-f	1	14725.082^a^	21799.783^b^	0.067^b^	15.740^a^
	28	13664.791^b^	20176.062^c^	0.073^a^	15.406^b^
	56	14982.826^a^	22405.761^a^	0.068^b^	15.723^a^

The Chao1, ACE, Simpson and Shannon indexes are presented for a similarity of 0.97 between the reads. Values of the same group within a column with different superscript letters mean the index differ significantly at different time points (*P* < 0.05, n =3).

#### Principal component analysis of the gut microbiota

The sequences of 36 fecal samples from 1, 28 and 56 d were used for principal component analysis. The similarity between microbiota can be expressed by the BeTa diversity analysis using the unweighted UniFrac and QIIME procedure. Each point represents one sample’s microbiota, and the distance between points represents the similarity between sequences of two samples’ microbiota.

We found that the gut microbiota at the three tested time points were separated from each other, except for one of the 28 d microbiota, and that the microbiota gathered together at the same time point (see Additional file 2: Figure S2), showing that the gut microbiota in rats was significantly different at the different time points and treatments. The gut microbiota in the control group at 28 d was not separated from the 1 d microbiota, indicating that the normal diet did not affect the gut microbiota at 28 d, and the abundance and diversity of the gut microbiota had not changed significantly (Table 1).

#### Composition of the gut microbiota

**Figure 3:**
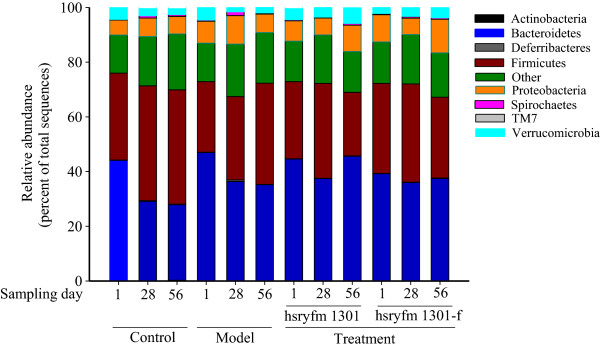
**Relative abundance of the gut microbiota in rats at the phylum level at different time points.** Control group: normal diet; model group: high-fat diet; hsryfm 1301 group: high-fat diet + *L. rhamnosus* hsryfm 1301-containing skim milk suspension; hsryfm 1301-f group: high-fat diet + *L. rhamnosus* hsryfm 1301-containing skim fermented milk.

The gut microbiota of the rats was constituted of Bacteroidetes, Firmicutes, Proteobacteria, Spirochaetes and Verrucomicrobia at the phylum level, and the Bacteroidetes were the most abundant, followed by Firmicutes and Proteobacteria (Figure 3). After being fed a normal or high-fat diet for 56 d, the relative abundance of Bacteroidetes and Verrucomicrobia in the rats’ gut significantly decreased (*P* < 0.05) and of Firmicutes significantly increased (*P* < 0.05; Table 2). Except for Bacteroidetes, opposite results were observed after treatment with *L. rhamnosus* hsryfm 1301 or its fermented milk for 28 d (*P* < 0.05; Table 2). The relative abundance of Spirochaetes in the control, hsryfm 1301 and hsryfm 1301-f groups significantly increased, whereas it significantly decreased in the model group at 56 d compared with that at 1 d (*P* < 0.05; Table 2).

**Table 2:** **Difference in abundance of intestinal microbiota in rats**

	Relative abundance (percent of total sequences)							
	1 d				56 d			
Taxon	Control	Model	hsryfm 1301	hsryfm 1301-f	Control	Model	hsryfm 1301	hsryfm 1301-f
Actinobacteria	0.10 ± 0.01	0.17 ± 0.02	0.20 ± 0.01	0.13 ± 0.01	0.27 ± 0.01	0.17 ± 0.02	0.13 ± 0.01	0.13 ± 0.01
*Collinsella*	0.00 ± 0.00	0.10 ± 0.01	0.10 ± 0.01	0.06 ± 0.01	0.17 ± 0.02*	0.09 ± 0.001	0.10 ± 0.01	0.10 ± 0.01**
Bacteroidetes	44.00 ± 2.50	46.80 ± 1.56	44.40 ± 2.12	39.10 ± 1.20	27.63 ± 1.53**	35.03 ± 1.20**	45.50 ± 2.62	40.33 ± 1.72
*Bacteroides*	1.47 ± 0.21	2.43 ± 0.12	2.33 ± 0.15	1.93 ± 0.20	1.10 ± 0.16	1.40 ± 0.11*	4.50 ± 0.26**	3.33 ± 0.28**
*Barnesiella*	0.10 ± 0.01	0.07 ± 0.01	0.08 ± 0.02	0.08 ± 0.01	0.17 ± 0.01	0.10 ± 0.01	0.00 ± 0.00*	0.00 ± 0.00*
*Parabacteroides*	0.30 ± 0.02	0.47 ± 0.03	0.40 ± 0.02	0.33 ± 0.01	0.20 ± 0.01	0.30 ± 0.02*	0.45 ± 0.01*	0.40 ± 0.01
*Alistipes*	0.30 ± 0.02	0.10 ± 0.01	0.10 ± 0.01	0.13 ± 0.01	0.10 ± 0.01*	0.17 ± 0.01*	0.05 ± 0.01*	0.13 ± 0.01
*Prevotella*	20.47 ± 1.02	22.77 ± 1.13	18.93 ± 1.08	17.67 ± 1.12	16.10 ± 2.03*	14.83 ± 1.16**	21.03 ± 1.15*	17.57 ± 1.09
Deferribacteres	0.10 ± 0.01	0.10 ± 0.02	0.10 ± 0.01	0.10 ± 0.01	0.20 ± 0.01*	0.10 ± 0.01	0.10 ± 0.02	0.17 ± 0.02*
*Mucispirillum*	0.10 ± 0.01	0.10 ± 0.02	0.10 ± 0.01	0.10 ± 0.01	0.13 ± 0.01	0.10 ± 0.01	0.10 ± 0.02	0.17 ± 0.02*
Firmicutes	31.80 ± 2.05	25.83 ± 1.53	28.20 ± 1.74	32.90 ± 1.85	41.80 ± 2.05*	37.03 ± 1.64*	23.23 ± 1.26*	27.57 ± 1.02*
*Lactobacillus*	0.10 ± 0.02	0.10 ± 0.01	0.10 ± 0.01	0.10 ± 0.02	0.12 ± 0.01	0.10 ± 0.02	0.17 ± 0.01*	0.27 ± 0.02*
*Butyrivibrio*	0.13 ± 0.01	0.15 ± 0.01	0.07 ± 0.01	0.10 ± 0.01	0.15 ± 0.01	0.10 ± 0.01	0.20 ± 0.02*	0.27 ± 0.02**
*Dorea*	0.13 ± 0.02	0.14 ± 0.01	0.17 ± 0.02	0.17 ± 0.02	0.09 ± 0.01	0.23 ± 0.01*	0.12 ± 0.01*	0.10 ± 0.01*
*Enterococcus*	0.10 ± 0.01	0.10 ± 0.01	0.10 ± 0.01	0.10 ± 0.01	0.15 ± 0.01*	0.37 ± 0.01**	0.05 ± 0.01*	0.06 ± 0.01*
*Oscillibacter*	1.63 ± 0.14	1.60 ± 0.11	2.07 ± 0.16	2.60 ± 0.21	2.33 ± 0.18*	3.37 ± 0.31**	1.43 ± 0.13*	1.83 ± 0.18*
*Ruminococcus*	0.30 ± 0.02	0.10 ± 0.01	0.20 ± 0.01	0.20 ± 0.01	0.57 ± 0.04**	0.37 ± 0.04**	0.16 ± 0.02*	0.17 ± 0.02*
*Allobaculum*	0.20 ± 0.01	0.27 ± 0.02	0.20 ± 0.01	0.20 ± 0.01	0.24 ± 0.02	0.10 ± 0.01*	0.47 ± 0.03**	0.27 ± 0.02
*Acetanaerobacterium*	0.00 ± 0.00	0.00 ± 0.00	0.07 ± 0.01	0.00 ± 0.00	0.10 ± 0.01*	0.00 ± 0.00	0.10 ± 0.01	0.03 ± 0.001*
*Holdemania*	0.00 ± 0.00	0.07 ± 0.01	0.00 ± 0.00	0.00 ± 0.00	0.00 ± 0.00	0.00 ± 0.00*	0.10 ± 0.01*	0.03 ± 0.01*
Proteobacteria	5.50 ± 1.05	8.03 ± 1.62	7.43 ± 1.16	10.00 ± 1.78	6.40 ± 0.76	6.80 ± 0.85	9.67 ± 1.13	12.30 ± 1.19
*Campylobacter*	0.13 ± 0.01	0.13 ± 0.02	0.10 ± 0.01	0.10 ± 0.01	0.11 ± 0.01	0.12 ± 0.01	0.00 ± 0.00*	0.00 ± 0.00*
*Helicobacter*	0.97 ± 0.12	1.17 ± 0.19	0.77 ± 0.05	2.40 ± 0.19	1.27 ± 0.22	1.20 ± 0.15	1.13 ± 0.21	1.27 ± 0.16*
*Escherichia*/*Shigella*	0.13 ± 0.01	0.10 ± 0.01	0.07 ± 0.01	0.13 ± 0.01	0.10 ± 0.002	0.07 ± 0.01	0.00 ± 0.00*	0.03 ± 0.001*
Spirochaetes	0.20 ± 0.02	0.43 ± 0.03	0.43 ± 0.02	0.33 ± 0.02	0.60 ± 0.03**	0.30 ± 0.02*	0.63 ± 0.04*	0.50 ± 0.02*
*Treponema*	0.20 ± 0.01	0.43 ± 0.03	0.43 ± 0.02	0.33 ± 0.02	0.40 ± 0.04**	0.30 ± 0.02*	0.60 ± 0.03*	0.50 ± 0.04*
Verrucomicrobia	4.30 ± 0.32	4.53 ± 0.36	4.50 ± 0.38	2.17 ± 0.23	2.63 ± 0.31*	2.00 ± 0.18**	5.73 ± 0.51*	3.73 ± 0.32*
*Akkermansia*	4.23 ± 0.30	4.50 ± 0.35	4.50 ± 0.38	2.13 ± 0.21	2.60 ± 0.29*	2.00 ± 0.18**	5.73 ± 0.51*	3.70 ± 0.25*
TM7	0.10 ± 0.01	0.00 ± 0.00	0.00 ± 0.00	0.10 ± 0.01	0.10 ± 0.01	0.10 ± 0.01*	0.00 ± 0.00	0.00 ± 0.00*
Other	13.90 ± 1.35	14.03 ± 1.42	14.77 ± 1.51	15.13 ± 1.47	20.40 ± 2.14*	18.47 ± 1.78	14.90 ± 1.65	16.20 ± 1.55

Each value is the mean ± standard deviation (n = 3). Data were analyzed using one-way ANOVA. Differences between treatment periods were assessed by the Duncan test. *means values in the same row of 56 d are significantly different from those of 1 d (*P* < 0.05); **, *P* < 0.01.

*Bacteroides* spp., *Prevotella* spp., *Oscillibacter* spp., *Helicobacter* spp. and *Akkermansia* spp. were the primary intestinal microbiota in the rats’ gut at the genus level (Table 2). The relative abundance of 22 genera of the intestinal microbiota belonging to Actinobacteria, Bacteroidetes, Deferribacteres, Firmicutes, Proteobacteria, Spirochaetes and Verrucomicrobia was significantly different after being fed a high-fat diet for 56 d and administered intragastrically *L. rhamnosus* hsryfm 1301 or its fermented milk for 28 d (*P* < 0.05; Table 2). The serum lipids in the control, hsryfm 1301 and hsryfm 1301-f groups were also significantly different compared with the model group at 56 d (*P* < 0.05; Figure 2D).

*L. rhamnosus* hsryfm 1301 and its fermented milk significantly decreased the relative abundance of *Barnesiella* spp., *Dorea* spp., *Enterococcus* spp., *Oscillibacter* spp., *Ruminococcus* spp., *Campylobacter* spp. and *Escherichia*/*Shigella* spp. (*P* < 0.05), while they significantly increased the relative abundance of *Bacteroides* spp., *Lactobacillus* spp., *Butyrivibrio* spp., *Holdemania* spp., *Treponema* spp. and *Akkermansia* spp. (*P* < 0.05; Table 2). *L. rhamnosus* hsryfm 1301 significantly increased the relative abundance of *Parabacteroides* spp., *Prevotella* spp., *Allobaculum* spp. and *Psychrobacter* spp. (*P* < 0.05), while it significantly decreased that of *Alistipes* spp. (*P* < 0.05; Table 2). *L. rhamnosus* hsryfm 1301 fermented milk significantly increased the relative abundance of *Collinsella* spp., *Mucispirillum* spp. and *Acetanaerobacterium* spp. (*P* < 0.05), while it significantly decreased the relative abundance of *Helicobacter* spp. (*P* < 0.05; Table 2).

## Discussion

Recently, it has been reported that the host intestinal microbiota depends not only on hereditary factors, but also on environmental factors including age, diet and living environment [16]. Diet intervention is controllable, and can improve the intestinal microbiota for the long-term and induce beneficial changes [17]. Our findings provide evidence for an important modification of the intestine resulting from a probiotic treatment, and indicate its contribution to improvement of host serum lipids.

Secondary bile acid, hydrogen sulfide and other harmful products are produced in rats during lipid metabolism after a high-fat diet intervention, which harm the colorectal mucosa and damage the micro environment of the intestine that helps bacteria survive [18, 19]. Consistently, we showed that high-fat diet decreased the abundance and diversity of the intestinal microbiota in rats, and that the abundance of Bacteroidetes and Firmicutes decreased and increased, respectively, in rats’ gut. However, some polysaccharides, bile acid and steroids in the diet could be absorbed and metabolized by Bacteroidetes, which the host cannot metabolize [20], and the calories in the food could be absorbed by Firmicutes, which promotes fat storage in the host body [21]. The relative abundance of *Akkermansia* spp. also decreased after a high-fat diet, which is consistent with Everard *et al*. [22]. *Akkermansia* spp. can improve the gastrointestinal mucosal barrier by increasing the rat gastrointestinal mucus layer thickness, and thus prevent some harmful substances from passing through the intestine into the blood, forestall fat mass storage *in vivo* and ameliorate high serum lipid-related metabolic diseases caused by obesity [22].

Lactic acid bacteria can compete for intestinal nutrients, and occupy some adsorption sites and metabolites to improve the intestinal environment in rats [23, 24]. Consistent with Xie *et al*. [25], the harmful bacteria *Escherichia*/*Shigella* spp. and the probiotic *Lactobacillus* spp. were suppressed and promoted, respectively, in the rats’ gut after administering intragastrically *L.rhamnosus* hsryfm 1301 or its fermented milk for 28 d. The rats’ serum lipids and efficiency of diet utilization improved by *L. rhamnosus* hsryfm 1301 or its fermented milk, which also recovered the abundance and diversity of the intestinal microbiota, which showed increased and decreased abundance of Bacteroidetes and Firmicutes, respectively. The short chain fatty acids produced by the recovered intestinal microflora reduced the TG and TC level by inhibiting the hepatic lipogenic enzyme activity and regulating the cholesterol distribution in the blood and liver [26, 27]. Accordingly, *L. rhamnosus* hsryfm 1301 and its fermentation products kept the balance of intestinal microbiota in rats, to alleviate the adverse effects induced by a high-fat diet.

Significant differences were observed in 22 genera of intestinal bacteria in the hsryfm 1301 and its fermented milk groups (Table 2). In fermented milk, not only the *Lactobacillus* itself, but also the fermentation products can have probiotic effects on the intestinal microbiota [28]. Recently, a study has shown that fermented milk could increase the number of cells with cytokines, which reduce cell death and enhance the function of the thymus to improve the mucosa and immune system of the host, thus balancing the gut ecology [28]. On the other hand, the fermented milk could improve the expression of the microbiome, which participates in lipid and carbohydrate metabolism [29]. The TC and LDL-C levels in the hsryfm 1301-f group were lower than those in the hsryfm 1301 group, possibly because the *L. rhamnosus* hsryfm 1301 fermented milk could enhance the expression of bacteria that improve lipid metabolism, such as *Butyrivibrio* spp. (Table 2).

Analysis of the association between intestinal microbiota and serum lipids at 56 d showed a positive correlation between bacteria related to *Ruminococcus* spp. and TG, *Dorea* spp. and TC and LDL-C, and between *Enterococcus* spp. and HDL-C. The positive correlation between *Ruminococcus* spp. and *Dorea* spp., which belong to Clostridium, and serum lipids (*P* < 0.05; Table 3), is consistent with the findings by Lahti *et al*. [30], who found that *Ruminococcus* spp. was enriched by polyunsaturated and odd-chain fatty acids, which are not synthesized in the body, and that *Ruminococcus* spp. facilitates the absorption of polyunsaturated dietary lipids. The bacteria related to *Butyrivibrio* spp. negatively correlated with TC and LDL-C (*P* < 0.05; Table 3), and *Butyrivibrio* spp. has the c9, tll activity of linoleate isomerase [31], which could decrease the mRNA expression of *SREBP*-*1c* [32]. Sterol regulatory element-binding protein (SREBP)-1c is one of the important elements that adjusts lipid metabolism [33, 34], thus the increase in abundance of *Butyrivibrio* spp. could lower the lipid levels in rats.

**Table 3:** **Associations between serum lipids and intestinal microbiota**

	TC	LDL	HDL	***Dorea***spp.	***Butyrivibrio***spp.	***Enterococcus***spp.	***Ruminococcus***spp.
TG	0.745	0.65	0.349	−0.305	−0.344	−0.422	0.982*
TC		0.799	0.778	0.951*	−0.646*	0.334	0.06
LDL			0.592	0.988*	−0.747*	0.109	0.025
HDL				−0.122	−0.408	0.953*	0.471
*Dorea* spp.					0.506	−0.175	0.41
*Butyrivibrio* spp.						−0.897	0.144
*Enterococcus* spp.							0.751

The SPSS Pearson analysis was used to calculate the Pearson’s correlation coefficient (r). *, *P* < 0.05. The sample sequencing of rats corresponds with the serum lipids of rats at 56 d (n = 3).

## Conclusions

We suggest that the intestinal microbiota and serum lipids in rats improved by feeding *L. rhamnosus* hsryfm 1301 or its fermented milk for 28 d. The correlation between serum lipids and *Ruminococcus* spp., *Dorea* spp. and *Enterococcus* spp. was positive (*P* < 0.05), while the correlation between *Butyrivibrio* spp. and serum lipids was negative (*P* < 0.05). We believe that long-term continuous monitoring of changes in intestinal microbiota will provide further insight into the role of intestinal microbiota in human lipid metabolism.

## Electronic supplementary material

Additional file 1: Figure S1: Rarefaction curve. Rarefaction results based on operational taxonomic unit (OTUs; 97% similarity). A, B, C and D indicate the control, model, hsryfm 1301 and hsryfm 1301-f group, respectively, and 1, 4 and 8 indicate sampling day 1, 28 and 56, respectively. (JPEG 66 KB)

Additional file 2: Figure S2: Principal component analysis of the rats’ gut microbiota at different time points. Before the study, the rats were fed a normal diet. From day 1, the rats were fed a high-fat diet, except for the control group, until day 28. After 28 days, the control group was fed a normal diet, the model group was fed a high-fat diet, the hsryfm 1301 group was fed a high-fat diet + *L. rhamnosus* hsryfm 1301-containing skim milk suspension, and the hsryfm 1301-f group was fed a high-fat diet + *L. rhamnosus* hsryfm 1301-containing fermented skim milk for 28 d. (JPEG 36 KB)

## Abbreviations

WHO: World Health Organization
CFU: Colony-Forming Units.
