# Supplementary figures and images for: The effect of Lactobacillus rhamnosus hsryfm 1301 on the intestinal microbiota of a hyperlipidemic rat model
Source: BMC Complement Altern Med. 2014 Oct 10;14:386. doi: 10.1186/1472-6882-14-386 (PMC4197344; doi:10.1186/1472-6882-14-386)

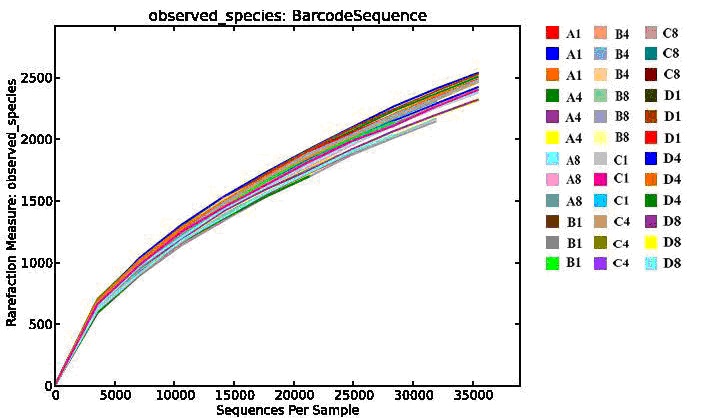

Supplement: Supplementary file 1 — Additional file 1: Figure S1: Rarefaction curve. Rarefaction results based on operational taxonomic unit (OTUs; 97% similarity). A, B, C and D indicate the control, model, hsryfm 1301 and hsryfm 1301-f group, respectively, and 1, 4 and 8 indicate sampling day 1, 28 and 56, respectively. (JPEG 66 KB) [file 12906_2014_1953_MOESM1_ESM.jpeg]

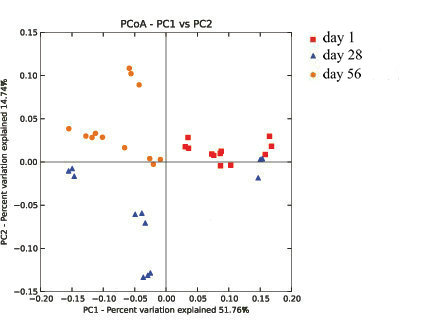

Supplement: Supplementary file 2 — Additional file 2: Figure S2: Principal component analysis of the rats’ gut microbiota at different time points. Before the study, the rats were fed a normal diet. From day 1, the rats were fed a high-fat diet, except for the control group, until day 28. After 28 days, the control group was fed a normal diet, the model group was fed a high-fat diet, the hsryfm 1301 group was fed a high-fat diet + L. rhamnosus hsryfm 1301-containing skim milk suspension, and the hsryfm 1301-f group was fed a high-fat diet + L. rhamnosus hsryfm 1301-containing fermented skim milk for 28 d. (JPEG 36 KB) [file 12906_2014_1953_MOESM2_ESM.jpeg]
